# Effectual Treatment of Acute Alcoholism

**Published:** 1896-03

**Authors:** 


### Effectual Treatment of Acute Alcoholism.

A young wife had just settled in her new home. All seemed fair and promising, but one night her husband came home very late and staggered into the house. His wife was greatly shocked, told him he was ill and to lie down at once. He did so. His face was reddish-purple, his breathing heavy, and altogether he was a pitiable-looking object. Mustard plasters were applied to his hands and feet. When the doctor came, felt his pulse, examined him, and found that he was drunk, he said:

"He will be all right in the morning."

But the wife insisted that he was very ill and severe remedies must be used.

"You must shave his head and apply blisters," she urged, "or I shall send for some one who will."

His head was accordingly shaved closely and blisters applied. All night he lay in a drunken sleep, notwithstanding the blisters.

About daylight he awoke to a most uncomfortable consciousness of blistered agonies.

"What does this mean?" he said, putting his hand to his bandaged head.

"Lie still; you mustn't stir," said the wife. "You have been very ill."

"I am not ill."

"Oh, yes, you are; you have brain fever. We have worked hard with you all night."

"I should think you had," groaned the victim. "What's the matter with my feet?"

"They are blistered."

"I am better now. Take off the blisters; do," he pleaded, piteously.

He was most uncomfortable; his head covered with sores and his hands and feet still worse.

"My dear," he said, groaning, "if I should get sick in this way again, do not be alarmed or send for a doctor, and, above all, do not blister me again."

"Oh, indeed, I will. All that saved you were the blisters; and if you should have another spell, I should be more frightened than ever, for the tendency, I am sure, is to apoplexy; and from the next attack you will be likely to die unless the severest measures are used."

From that day he has never had another attack of drink.—*Dover Journal-Annals of Hygiene.*

